# Bioassay-guided Isolation of Flavonoids from *Caesalpinia bonduc* (L.) Roxb. and Evaluation of Their Cytotoxicity

**DOI:** 10.22037/ijpr.2020.112557.13824

**Published:** 2021

**Authors:** Narges Pournaghi, Farahnaz Khalighi-Sigaroodi, Elahe Safari, Reza Hajiaghaee

**Affiliations:** a *Medicinal Plants Research Center, Institute of Medicinal Plants, ACECR, Karaj, Iran. *; b *Department of Immunology, School of Medicine, Iran University of Medical Sciences, Tehran, Iran.*

**Keywords:** Caesalpinia bonduc, Bioassay-guided, Fabaceae, Flavonoid, Phenol, Antioxidant activity, MTT assay

## Abstract

Cancer is one of the most important causes of death all around the world. Screening plants and their secondary metabolites as cytotoxic agents is one of the common methods for identifying new compounds used in chemotherapy and inhibition cancer process. *Caesalpinia bonduc *(L.) Roxb. from the Fabaceae family was used for improving wound, fever, tumor, hydrocele, hernia, smallpox, toothache, inflammation, and as astringent, anthelmintic, antidiabetic, and antimalarial agent in traditional medicine. A bioassay-guided study of this species led to the isolation of three flavonoids. At first, the cytotoxicity of methanol extract of aerial parts (leaves and stems), seeds, and legumes of this plant was tested against MCF-7 and PC-3 by MTT assay. The methanol extract of legumes showed better inhibitory activities (IC_50_ < 500 µg/mL). As a result, this extract was selected for fractionation. In the next step, the ethyl acetate (EtOAc) fraction was selected for phytochemical analysis based on the inhibitory activity (IC_50_ = 170 ± 0.9 µg/mL). In this way, total phenol content (625 ± 7.2 GAE/g extract) and antioxidant activity (IC_50_ = 6.1 ± 0.3 µg/mL) was compared by BHT (IC_50_ = 13.5 ± 0.7 µg/mL). Finally, three compounds including, quercetin-3-methyl ether (**1**), kaempferol (**2**), and kaempferol-3-O-α-L-rhamnopyranosyl-1→2)-β-D-xylopyranoside (**3**) were isolated from EtOAc fraction, and all isolated compounds were tested for their cytotoxicity and compound **1** showed better inhibitory activity than other two compounds. This study suggests that *Caesalpinia bonduc *could be considered for further investigations as a natural source of biological compounds.

## Introduction

Cancer is the second leading cause of death all around the world. About 10 million people are diagnosed with cancer each year, half of whom die (1, 2). The most common and deadly cancers in women and men are breast cancer and prostate cancer, respectively (3-5). The prevalence of these cancers has increased significantly (6). Medicinal plants are better sources of anti-cancer drugs than chemical drugs due to their low side effects (7). The choice of these plants is usually based on ethnobotanical resources or traditional medicine (8). Screening plants and their phytochemicals as natural killer agents for cancer cells is one of the most common methods for identifying new compounds used in chemotherapy and inhibiting the cancer process. For example, several species of the Fabaceae family have been studied for their cytotoxic effects (9-12). Also, in a review article, we expressed a number of compounds and properties of different species of the Genus *Caesalpinia* particularly cassane and norcassane compounds that have cytotoxicity effects (13).

*Caesalpinia bonduc *(L.) Roxb. (Fabaceae family), the accepted name instead of *Caesalpinia bonducella*, *Guilandina bonduc, *and *Guilandina bonducella*, is one of the *Caesalpinia* species represented in the flora of Iran (14, 15). This species is grown in tropical and subtropical regions (like India, Pakistan). In Iran, this plant is found in the South and Southeast of Iran (16). This species was used for improving wound, fever, tumor, hydrocele, hernia, smallpox, toothache, inflammation and as astringent, anthelmintic, antidiabetic, and antimalarial agent in traditional medicine (17). Many studies have been done on this plant based on traditional medicine sources. For example, the antibacterial effect of seed methanol extract (18), the antidiabetic effect of several extracts of seed (19), antitumor and antioxidant effect of leaves methanol extract on mice (20) and also antipsoriasis and increasing uterine smooth muscle contraction effects of leaves extract (21, 22) have been proven. Also, several components were separated from this plant like caesalls H-M (23), caesalpinolide C-E (24), caesalpinianone, 6-O-methylcaesalpinianone, stereochenol A, hematoxylol, 4’-O-acetylloganic acid, 6’-O-acetylloganic acid and 2-O-β-D-glucosyloxy-4-methoxybenzenepropanoic acid (25), bonducellipins A-D (26), 7-hydroxy-4’-methoxyl-3,11-dehydrohomoisoflavanone, 4,4’-dihydroxy-2’-methoxy-chalcone, 7,4’-dihydroxy-3,11-dehydrohomoisoflavanone, luteolin and kaempferol-3-O-β-D-xylopyranoside (27). In another study, cytotoxicity of methanol extracts of different parts (legume, seed, and aerial part) of *Caesalpinia bonduc (C. bonduc)* was tested through the brine shrimp lethality assay. The legume extract of *C. bonduc* showed significant cytotoxicity (28). According to the evidence of the antitumor effect of methanol extract of this plant, the aim of this study was to investigate the anti-cancer effect of extracts and different parts of this plant and to isolate the compounds responsible for this property.

## Experimental

*General*

Nuclear magnetic resonance (NMR; 500 MHz) spectra were recorded on Bruker FT-500 spectrometer instrument using DMSO-d_6_ solvent and TMS (tetramethylsilane) as an internal standard. The mass spectra of the compounds were obtained by Agilent Technologies mass spectrometer Model: 5975C VL MSD with Triple-Axis Detector (70 eV). All reagents and chemicals for phytochemical experiments and MTT assay were analytical grades.

*Plant material*

The aerial parts (leaves and stems), seeds, and legumes of *Caesalpinia bonduc* (L.) Roxb. were collected from the Sarbaz region (Sistan and Baluchestan Province) in the southeast of Iranian July 2014. The plant was identified in the Institute of Medicinal Plants Herbarium (MPHI.IR).

*Extraction and Isolation*

The experiment was accomplished in several steps. In the first step, air-dried and powdered legumes (1500 g), seeds (2000 g), and aerial parts (840 g) were extracted at room temperature with 9000 mL, 10130 mL, and 8170 mL methanol by percolation method, respectively. Then these extracts were concentrated by a rotary evaporator (Heidolph Laborota 4000 eﬃcient) and dried. The extraction yields were 4.13% w/w (legume), 4.75% w/w (seed) and 13.55% w/w (aerial parts). Then MTT assay was performed on these extractions and the legume extraction was selected for fractionations based on the MTT results.

In the next step, the methanol extract of legume was dispersed in distilled water and partitioned with n-hexane, chloroform (CHCl_3_), ethyl acetate (EtOAc), and n-butanol (n-BuOH) consecutively based on increasing the polarity of solvents to gain n-hexane, CHCl_3_, EtOAc, n-BuOH, and water-soluble fractions. Based on the results of different evaluations, including total phenol content, DPPH radical scavenging assay and the MTT assay, the EtOAc soluble fraction was selected for more fractionations and purification.

**Figure 1:**
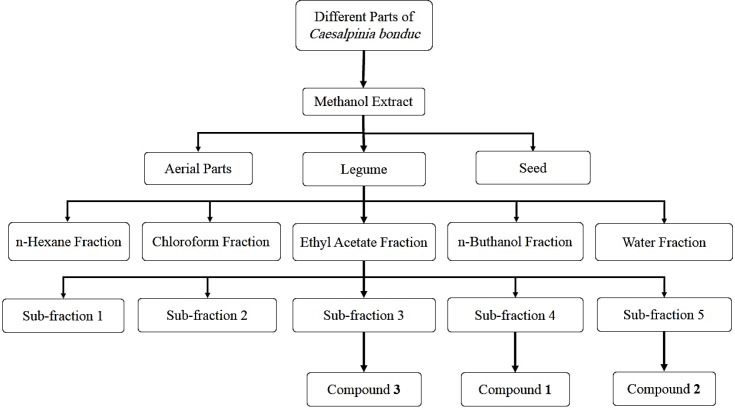
Schematic procedures for extraction and fractionation

In the final step, 4 g of the EtOAc soluble fraction was fractionated by the Sephadex LH-20 column chromatography and MeOH as eluent. Five sub-fraction (1-5) were gained. Sub-fraction 4 (110 mg) and 5 (40 mg) were purified by silica gel plate (20 × 20 cm) with chloroform-methanol (90:10) to get compound **1** (12.5 mg) and **2** (7 mg). Compound **3** (14 mg) was isolated from the sub-fraction 2 (300 mg) using silica gel column chromatography (70-230 mesh ASTM) with CHCl_3_-MeOH (97:3 to70:30). This compound was more purified on the Sephadex LH-20 column using MeOH as eluent (Figure 1). Compound structures were identified by ^1^H-NMR and ^13^C-NMR and MS spectral analysis, as well as by comparing with the data published in the literature. Then the cytotoxicity of isolated compounds was determined by MTT assay.

*Cell culture*

MCF-7 (human breast cancer), PC-3 (human prostate cancer), and HepG-2 (Human liver cancer) were purchased from the Pasture Institute of Tehran (Tehran, Iran). These cell lines were cultured in RPMI (Roswell Park Memorial Institute) medium containing 10% FBS (Fetal Bovine Serum), 100 IU/mL penicillin, and 100 µg/mL streptomycin under humidiﬁed atmosphere at 37 °C in a 5% CO_2_ incubator.

*MTT assay*

Antiproliferative activity of extracts, fractions, and isolated compounds was measured by MTT assay using MCF-7, PC-3, and HepG-2 cancer cell lines (29). Methotrexate was used as a positive control. Ten-thousand cells per well (at the exponential growth phase) were seeded into a flat bottom 96-well plate. After 24 h incubation in a 5% humidified CO_2_ incubator at 37 °C, the cells were treated with a fresh medium containing different concentrations of extracts, fractions, and pure compounds in triplicates. After 48 and 72 h of incubation at 37 °C, 10 μL/well MTT (3-(4,5-dimethyl thiazolyl)-2,5-diphenyltetrazolium bromide: stock solution 5 mg/mL PBS) was added and the plate was again incubated at 37 °C for 4 h. 100 μL DMSO was added to each well and shaking for 15 min. The absorbance was recorded at 630 nm using a microplate reader and IC_50_ values for each cell line were measured.

*Total phenol content*

The total phenol content of the fractions was measured by the Folin-Ciocalteu method (30). First, 100 µg/mL concentration of the fraction solutions was prepared and oxidized with Folin-Ciocalteu solution followed by neutralization with 7% (w/v) sodium carbonate. After shaking for 2 h at room temperature, the absorption was measured at 765 nm. Gallic acid was used as a standard and its solutions (50, 100, 150, 500, and 1000 µg/mL) were prepared to draw a calibration curve. All standard and fractions were performed in triplicate and total phenol content was expressed as Gallic acid equivalent (GAE)/g extract.

*DPPH radical scavenging assay*

The Free radical-scavenging activity of fractions was evaluated by the 2,2-Diphenyl-1-picrylhydrazyl (DPPH) (31). Brieﬂy, diﬀerent concentrations of fractions (5, 10, 20, 40, and 80 µg/mL) and 40 µg/mL solution of DPPH in methanol were prepared. In each well of 96-well plates, 63 µL of DPPH was added to a suitable volume of each fraction solution until the final volume became 250 µL. The absorbance was measured at 517 nm after 1 h. The test was performed in triplicate. DPPH solution alone served as control and butylated hydroxytoluene (BHT) was used as standard.

## Results and Discussion

All processes of the experiment contained phytochemical process followed by biological assays. In the first step, the methanol extracts of aerial parts, seeds and legumes of *C. bonduc *were prepared and tested their cytotoxicity.

*Antiproliferative activity of the legume, seed, and aerial part extracts*

MTT assay was applied to determine which extract had better antiproliferative activity and fewer IC_50_. The results showed that the MeOH extract of legumes had the least IC_50_ value of 483 and 337 µg/mL against MCF-7 and PC-3, respectively. Therefore, it was selected for further fractionation (Table 1).

In the next step, n-hexane, chloroform, ethyl acetate, n-butanol and water soluble fractions were fractionated from the methanol extract of legumes. Total phenol content, antioxidant activity, and antiproliferative activity of fractions were measured to determine effective fraction for phytochemical analysis.

*Total phenol content of fractions*

Total phenol contents of *C. bonduc* fractions and methanol extract of legume were determined and expressed as Gallic acid equivalent (GAE)/g. Total phenol content varied between 175 for water fraction and 625 for ethyl acetate fraction (Table 2). As shown in Table 2, ethyl acetate fraction has the highest amount of phenol content (625 GAE/g).

*DPPH radical scavenging assay*

The antioxidant activity of fractions was determined through DPPH free radical scavenging activity test. The results revealed that ethyl acetate fraction has higher ability for radical scavenging activity (IC_50_: 6.1 µg/mL) compared to BHT (IC_50_: 13.5 µg/mL) (Table 2).

According to the results, a comparison of total phenol contents of fractions with their DPPH radical scavenging activity shows that there is a direct relationship between the total phenol compound and antioxidant activity of fractions.

*Antiproliferative activity of fractions*

The methanol extract of legumes and all fractions were tested for their cytotoxicity against three human cancer cell lines (MCF-7, HepG-2, and PC-3) using the MTT assay. The IC_50_ values were measured (Table 3). Ethyl acetate fraction showed better activity against all of the tested cell lines. It should be noted that we used 72 h of incubation at 37 °C in this test because it had better response in previous test.

According to the results listed above (total phenol content, DPPH radical scavenging assay and antiproliferative activity of fractions), the ethyl acetate fraction was selected for more fractionations and purification.

**Figure 2:**
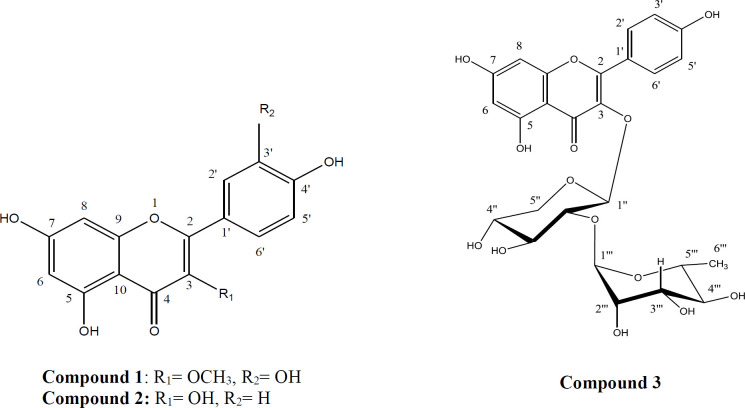
Chemical structures of compounds** 1-3** isolated from *Caesalpinia bonduc*

In the final step, from the bioactive guided fractionation of ethyl acetate fraction of *C. bonduc* legume, three flavonoids (compounds **1** to **3**) were obtained. Fractionation and purification of ethyl acetate fraction were performed by the Sephadex LH-20 column chromatography and silica gel plate chromatography. The structures of the isolated compounds were characterized as Quercetin-3-methyl ether (**1**), Kaempferol (**2**), Kaempferol-3-O-α-L-rhamnopyranosyl-1→2)-β-D-xylopyranoside (**3**) using ^1^H-NMR, ^13^C-NMR, and MS evaluations, as well as comparison with those reported in the literature (32, 33 and 27). Figure 2 illustrates the chemical structures of compounds **1**-**3**.

*Spectroscopic data of the isolated compounds*

Compound** 1:** Quercetin-3-methyl ether (C_16_H_12_O_7_); pale yellow crystalline solid; ^1^H-NMR (500 MHz, DMSO-d_6_); δ 12.70 (1H, 5-OH), 7.54 (1H, *d*, *J *= 2.2 Hz, H-2′), 7.45 (1H, *dd*, *J =* 8.35, 2.2 Hz, H-6′), 6.91 (1H, *d*, *J *= 8.35 Hz, H-5′), 6.39 (1H, *d*, *J *= 2.00 Hz, H-8), 6.18 (1H, *d*, *J *= 2.00 Hz, H-6), 3.78 (3H, *s,*3-OCH_3_);^13^C-NMR (125 MHz, DMSO-d_6_); δ 178.21 (C-4), 165.21 (C-7), 161.65 (C-5), 156.80 (C-9), 155.93 (C-2), 149.28 (C-4′), 145.73 (C-3′), 138.11 (C-3), 121.13 (C-1′), 120.99 (C-5′), 116.18 (C-2′), 115.74 (C-6′), 104.35 (C-10), 99.14 (C-6), 94.11 (C-8), 60.09 (OCH_3_); EI-MS *m/z*: [M+H]^+^ 317 (32,27).

Compound** 2:** Kaempferol (C_15_H_10_O_6_); pale yellow powder; ^1^H-NMR (500 MHz, DMSO-d_6_); δ 12.73 (1H, 5-OH), 8.10 (2H, *d*, *J *= 9.7 Hz, H-2′, 6′), 6.92 (2H, *d*, *J =* 9.7 Hz, H-3′, 5′), 6.79 (1H, br s, H-8), 6.25 (1H, br s, H-6); ^13^C-NMR (125 MHz, DMSO-d_6_); δ 180.36 (C-4), 164.92 (C-7), 162.00 (C-5), 161.63 (C-4′). 157.90 (C-9), 149.50 (C-2), 135.21 (C-3), 130.12 (C-2′, 6′), 121.45 (C-1′), 116.31 (C-5′, 3′), 103.52 (C-10), 99.70 (C-6), 94.50 (C-8) (33). 

Compound** 3:** Kaempferol-3-O-α-L-rhamnopyranosyl-1→2)-β-D-xylopyranoside (C_26_H_28_O_14_); pale yellow crystalline solid; ^1^H-NMR (500 MHz, DMSO-d_6_); 7.98 (2H, *d*, *J *= 8.85 Hz, H-2′,6′), 6.89 (2H, *d*, *J =*8.35 Hz, H-3′,5′), 6.17 (1H, br s, H-8), 5.97 (1H, br s, H-6), 5.52 (1H,* d*, *J *= 7.35 Hz, H-1′′), 5.09 (1H, s, H-1′′′); ^13^C-NMR (125 MHz, DMSO-d_6_); δ 176.84 (C-4), 167.05 (C-7), 161.40 (C-5), 160.83 (C-4′). 157.08 (C-9), 155.27 (C-2), 132.59 (C-3), 130.77 (C-2′,6′), 121.04 (C-1′), 115.74 (C-5′,3′), 107.01 (C-10), 102.30 (C-1′′′), 101.10 (C-1′′), 99.69 (C-6), 88.70 (C-8), 77.60 (C-2′′), 77.35 (C-3′′), 72.31 (C-4′′), 70.99 (C-2′′′), 70.18 (C-5′′′), 68.83 (C-5′′), 17.89 (C-6′′′); EI-MS *m/z*: [M+H]^+^565 (32, 27).

*Antiproliferative activity of isolated compounds*

The isolated compounds were tested for antiproliferative activity against MCF-7 (human breast cancer), HepG-2 (human liver cancer) and PC-3 (human prostate cancer). The results of the *in-vitro* antiproliferative activity of the compounds isolated from *C. bonduc* are summarized in Table 4.

The results show that compound** 1** (Quercetin 3- methyl ether) has moderate antiproliferative activity with IC_50_ values of 45 µg/mL against PC-3, but it showed low antiproliferative activity with IC_50_ values of 78 and 99 µg/mL against MCF-7 and HepG-2, respectively. Previous investigation has proven the cytotoxicity of compound **1** against Hela cell line and mouse epidermal JB6 P1 cells (27, 34). Compounds **2** and **3** did not show any signiﬁcant antiproliferative activity, and both had IC_50_ values greater than 100 µg/mL. The lack of cytotoxic activity of compound **3** might be due to the additional sugar component attached at position 3 of the C-ring so that increasing their polarity and limiting their cellular permeability and also increased molecular weight of compound **3** might limit its cellular permeability (35). Due to the structure of the compounds and its relationship with antiproliferative activity, the presence of a hydroxyl group at 3’ of the B-ring and methylation of the 3 hydroxyl group at the C-ring are the factor for increasing this property.

It is necessary to mention that different flavonoid compounds were separated from this plant, for example caesalpinianone, 6-O- methylcaesalpinianone, 7-hydroxy-4’-methoxyl-3,11-dehydrohomoisoflavanone, 4,4’-dihydroxy-2’-methoxy-chalcone, 7,4’-dihydroxy-3,11-dehydrohomoisoflavanone, luteolin and kaempferol-3-O-β-D-xylopyranoside. Also the cytotoxicity of some of these compounds was investigated. 7,4’-Dihydroxy-3,11-dehydrohomoisoflavanone had antiproliferative activity against Hela and BGC 823 cells lines and also luteolin had antiproliferative activity against Hela cells lines (27).

Various mechanisms have been proposed for flavonoids cytotoxicity, including inhibition of DNA replication, activating path of apoptosis, inhibition of oxidative processes, and decreasing level of redox-active proteins (36-39). Other reports showed that polyhydroxylic flavonoids and quercetin inhibit the growth of cancer cells and reduce DNA production and prevent cell crossing from cell cycle G_1_ step to S step (40). In most of these investigations, there is a correlation between phenol compounds and the antioxidant capacity of the extracts and their effects on cancer cells. In this study, the biochemical pathways and the mechanism of action of EtOAc fraction and compound **1** in inhibition of cancer cells were not investigated. However, it was found that the extract has antioxidant compounds and there is a correlation between flavonoids and cancer growth inhibition.

**Table 1:** Antiproliferative activity of the methanol extracts on the cancer cell lines (Mean ± SD, n = 3).

**Extract**	**IC**_50_ ** (µg/mL)**			
	**MCF-7**		**PC-3**	
	**48 (h)**	**72 (h)**	**48 (h)**	**72 (h)**
Legume	546 ± 0.5	483 ± 1.2	456 ± 1.9	337 ± 1.1
Seed	>1000	>1000	800 ± 2.2	730 ± 4.2
Aerial part	>1000	850 ± 6.1	780 ± 5.3	510 ± 3.1

**Table 2:** Results of the total phenol content and DPPH IC_50_ (Mean ± SD, n = 3).

	**Methanol extract**	**n-Hexane fraction**	**Chloroform fraction**	**Ethyl acetate fraction**	**n-Butanol fraction**	**Water fraction**	**BHT**
GAE/g	475 ± 3.2	275 ± 2.4	575 ± 5.1	625 ± 7.2	300 ± 3.2	175 ± 1.8	-
DPPH IC_50_ (µg/mL)	14.3 ± 0.3	22.5 ± 0.9	7.7 ± 0.5	6.1 ± 0.3	17.2 ± 0.9	27.3 ± 1.1	13.5 ± 0.7

**Table 3:** *In-vitro* cytotoxic activities of fractions

**fractions**	**MCF-7**	**HepG-2**	**PC-3**
	**IC**_50_ ** ± SD** ^a ^ **(µg/mL)**	**IC**_50_ ** ± SD** ^a ^ **(µg/mL)**	**IC**_50_ ** ± SD** ^a ^ **(µg/mL)**
Methanol extract	483 ± 1.2	800 ± 3.2	337 ± 1.1
n-Hexane fraction	>1000	>1000	>1000
Chloroform fraction	333 ± 2.1	560 ± 0.9	300 ± 1.4
Ethyl acetate fraction	280 ± 1.3	191 ± 3.1	170 ± 0.9
n-Butanol fraction	700 ± 5.2	850 ± 3.4	540 ± 2.1
Water fraction	>1000	>1000	>1000

^a^Values presented mean ± SD of triplicate experiments.

**Table 4:** *In-vitro* cytotoxic activities of isolated compounds

**Isolated compounds**	**MCF-7**	**HepG-2**	**PC-3**
	**IC**_50_ ** ± SD** ^a ^ **(µg/mL)**	**IC**_50_ ** ± SD** ^a ^ **(µg/mL)**	**IC**_50_ ** ± SD** ^a ^ **(µg/mL)**
**1**	78 ± 0.8	99 ± 1.0	45 ± 0.5
**2**	> 100	> 100	> 100
**3**	> 100	> 100	> 100
methotrexate^b^	25.3 ± 1.2	8.5 ± 0.8	12.5 ± 1.0

^a^Values present mean ± SD of triplicate experiments.

^b^Positive control.

## Conclusion

The results of this study show that ethyl acetate fraction of *C. bonduc* legume has antioxidant properties and inhibition effects on cancer cell lines. These effects are related to the presence of secondary metabolites, especially flavonoids. Further studies are needed to determine all components with anti-cancer effects in *C. bonduc*.
